# Analysis of Energy Efficiency in WPC Production from *Pinus sylvestris* Wood and Thermoplastic ABS Supported by the HWE Method

**DOI:** 10.3390/ma18050980

**Published:** 2025-02-23

**Authors:** Kamil Roman, Katarzyna Fedorowicz

**Affiliations:** 1 Institute of Wood Sciences and Furniture, Warsaw University of Life Sciences, 166 Nowoursynowska St., 02-787 Warsaw, Poland; 2 Faculty of Wood Technology, Warsaw University of Life Sciences—SGGW, 159 Nowoursynowska Str., 02-776 Warsaw, Poland; 206516@sggw.edu.pl

**Keywords:** hot water extraction, lignocellulosic material, extraction, *Pinus sylvestris*, energy

## Abstract

This study evaluates the mechanical energy consumption involved in producing wood–plastic composites (WPC) using Scots pine (*Pinus sylvestris*) and a acrylonitrile–butadiene–styrene terpolymer (ABS) thermoplastic. The research examines the effects of Hot Water Extraction (HWE) on the properties of *Pinus sylvestris* biomass and its application in biocomposite production. Two *Pinus sylvestris* fractions, *f1* (0–1 mm) and *f2* (1–4 mm), were analyzed with and without HWE during compaction. The energy requirements and material performance were assessed through moisture content control, ash content determination, and compaction testing. The results show that HWE significantly improves the physical and chemical properties of *Pinus sylvestris*, increasing its suitability for WPC production. The HWE-treated samples consumed less energy and exhibited a higher density compared to the untreated materials. Statistical analysis validated the reliability of the methodology and revealed significant differences in the energy efficiency and material compatibility between treated and untreated samples. This study highlights the potential use of *Pinus sylvestris* and ABS for renewable bio-composite production, underlining the critical role of HWE in enhancing the properties of lignocellulosic materials. The findings contribute to developing energy-efficient industrial processes aligning with circular economy objectives.

## 1. Introduction

This study aims to evaluate the mechanical energy consumption involved in producing wood–plastic composites (WPC) using Scots pine (*Pinus sylvestris*) and a acrylonitrile-butadiene-styrene terpolymer (ABS) thermoplastic. The wood–plastic composites (WPC) possess unique properties such as durability, weather resistance, and versatility, making them highly suitable for various applications [1,2]. Despite this, it is still unclear how HWE can be used to optimize *Pinus sylvestris* for multiple applications. In the furniture industry, WPC can effectively replace traditional particleboard, offering enhanced strength and resistance to environmental factors. This makes it particularly well-suited for outdoor furniture and small architectural elements. WPC is increasingly used in dashboards, door panels, trims, and other interior components in the automotive sector. Its lightweight, durable, and easily moldable nature makes it a preferred material for manufacturers aiming to reduce vehicle weight and improve fuel efficiency. In Germany, WPC is particularly valued for its safety features, such as eliminating sharp edges and superior acoustic properties, which make it ideal for components like doors, rear seats, dashboards, and body panels. Similarly, WPC plays an essential role in the packaging industry due to its moisture resistance and moldability, which enable the creation of packaging for food, pharmaceuticals, and cosmetics [3].

The versatility of WPC extends far beyond its established uses, showcasing its ability to meet diverse regional and sector-specific demands. WPC provides manufacturers with a renewable and versatile production solution by combining the advantages of wood and thermoplastics. Its ease of processing and high moldability enable its customization to meet specific design and functional requirements across various industries. These unique properties address the growing demand for environmentally conscious alternatives and enhance manufacturing efficiency. The remarkable adaptability of WPC makes it an ideal choice for a wide range of industries, including construction, architecture, automotives, furniture, and sports, further driving the adoption of innovative and renewable materials.

Enhancing WPC properties through advanced treatment processes, particularly those that use heat, is one of the key factors contributing to their versatility and performance. In WPC, high-temperature methods, such as the HWE method and thermal modification, play an essential role in optimizing the structural and mechanical characteristics of the wood components. By improving their durability and resistance to environmental factors, these treatments also improve material compatibility, allowing wood fibers to be better integrated with thermoplastic materials like acrylonitrile–butadiene–styrene terpolymer (ABS) [4,5]. In the process, WPC is improved in demanding applications, from outdoor structures to automotive components. Thermal treatments, especially those involving heat, significantly impact wood’s chemical and physical properties [6]. Reducing the hemicellulose content and altering the lignin structure can improve mechanical properties like the tensile strength, flexibility, and dimensional stability, which are enhanced by heating processes between 160 and 260 °C [7]. Construction, furniture, and bio-composite applications require these modifications to ensure that *Pinus sylvestris* wood fibers are strong enough to withstand the demands of these applications. Innovative and renewable materials can be made using lignocellulosic raw materials from *Pinus sylvestris* using heat treatments like HWE [8]. Thermal modification techniques and the inherent versatility of WPC have been linked to these advancements, leading to environmentally friendly materials and a wider range of industrial applications. The purpose of this article is to present the materials and methods used in the study, present the findings of the analysis, discuss the findings, and finally, draw conclusions.

The high-temperature heat treatment of *Pinus sylvestris* wood alters its chemical and physical structure, improving its mechanical properties, durability, and resistance to biological agents [9]. It involves heating wood at high temperatures (160–260 °C) without oxygen. During this process, the wood hemicelluloses and lignin content is reduced, affecting its physical and mechanical properties, such as its hardness, dimensional stability, and moisture resistance. The cells in *Pinus sylvestris* wood include fibers and vessels with different functions [10,11]. Heat treatment alters the properties and relationships between these cells. Research focuses on the effects of heat treatment on the mechanical properties of *Pinus sylvestris* wood, such as its compressive strength [12,13]. Studies on *Pinus sylvestris* have also shown that HWE improves resistance to certain fungi, though not universally [9]. Prior studies have investigated the effects of HWE on *Pinus sylvestris* properties. Still, much less is known about its potential for optimizing *Pinus sylvestris* for various applications, such as biocomposite production.

## 2. Materials and Methods

### 2.1. Characteristics of the Research Material

The research was divided into key stages using appropriate methods and tools. The raw wood material was prepared using standard procedures, including shredding, fractionation, and drying, to ensure the homogeneity of the starting material. HWE then modified the material in a laboratory reactor with a heating jacket, followed by a key modification. The purpose of this step was to affect the wood’s chemical composition and physical properties. HWE-modified and control materials samples were compacted using a hydraulic press, forming samples of preset shapes for subsequent analysis. The material properties were characterized using a universal testing machine, focusing on the compressive strength. The study was further complemented by a microscopic analysis of the wood’s microstructure. These methods were selected based on the necessity of assessing the properties of *Pinus sylvestris* holistically from the microstructure to the micromechanical scale. To determine whether this material is suitable for biopolymer composites, it must be analyzed on a macro scale. Detailed descriptions of each methodological step are provided in the following subsections.

When creating WPC, *Pinus sylvestris* wood was used as a material naturally found in Europe and Asia [11]. This study obtained wood from ten *Pinus sylvestris* trunks from a managed plantation in the Supraśl Forest District, Poland. There were approximately 50-year-old trees in the forest. In this study, heartwood and sapwood were both used. The wood material was pre-chipped, fractionated in a lab mill, and sieved through vibrating screens at C.B.K.O Hydrolab (Warsaw, Poland). The wood was ground into two fractions of 0–1 mm and 1–4 mm before use to increase the polymer contact area and homogeneity. *Pinus sylvestris* wood tends to have a 500 kg/m^3^ density, assuming a 12% moisture content. Wood waste from sawmills and other wood industries, such as fine wood particles (0–1 mm and 1–4 mm), can be used in a circular economy to make it more sustainable. The laboratory tests used two fractions of *Pinus sylvestris* material and ABS plastic. Besides being strong, complex, and chemically resistant, ABS is one of the most desirable thermoplastic materials. We examined how *Pinus sylvestris* wood waste behaves regarding its granular properties. To prepare the material for chip testing, the diameter of the collected pieces was measured, as well as the moisture content and thickness of the raw material.

*Pinus sylvestris* was chosen for this study because it possesses several properties that make it a suitable candidate for biocomposite production. Its ease of processing makes it ideal for manufacturing WPC. The *Pinus sylvestri’s* inherent strength contributes to the materials’ structural integrity and durability. *Pinus sylvestris* is a readily available and cost-effective raw material that is important for manufacturing. Its wide use in various wood-based industries, such as furniture, construction, and wood-based panels, further supports its suitability for biocomposite applications. These factors collectively make *Pinus sylvestris* an ideal candidate for this research, allowing for a comprehensive evaluation of the energy efficiency and material performance of the biocomposite. Therefore, correctly choosing and preparing the raw wood materials before production is essential to ensure that they are properly removed.

An ABS polymer was used in these composites; this was a thermoplastic obtained by post-dimerizing [14]. This material is widely used in various industries, from automobile manufacturing to household appliance manufacturing, where high strength and an aesthetic performance are required. The characteristics of ABS include a high impact strength, stiffness, and abrasion resistance, as well as its ability to be recycled many times, which is particularly important from the point of view of waste minimization. The material is also chemically resistant, which makes it durable in various operating conditions. While ABS plastic has many advantages, its petroleum-derived origins make its environmental impact crucial to acknowledge. Responsible sourcing, recycling, and closed-loop practices are essential to mitigate these concerns. The recyclability of this plastic reduces its environmental impact and fits into the idea of a closed-loop economy. As a result of combining *Pinus sylvestris* wood and ABS, a WPC composite with improved properties, such as resistance to moisture and biological degradation, better dimensional stability, and excellent mechanical durability, was produced.

### 2.2. Physical Parameters of Wood

The wood’s anisotropic and hygroscopic nature significantly influenced its behavior within the WPC. Moisture content and density are crucial parameters that play a significant role in determining the properties of the final composite material. The moisture content of the wood was determined using the oven-dry method [15]. This method hinges on selecting the weight difference between the sample before and after drying. For this study, samples weighing approximately 5 g were used. The samples were dried at 103 °C in an electric oven with precise temperature control. Regular check scales were conducted throughout the drying process. Drying was considered complete when the weight difference between two consecutive scales was no more than 0.002 g. To calculate the moisture content of the wood, the difference in weight between the wet and dry states was divided by the initial weight of the wet sample and then multiplied by 100%. The results were rounded to the nearest 0.1%.

To determine the moisture content of finely ground wood, such as sawdust or wood chips, selecting a suitable sample size measured by weight is essential. The wood density, tree species, and moisture content are the most critical physical properties [16]. The tree species determines the anatomical structure, directly affecting the wood density. The apparent density of wood increases as its moisture content increases since water adds to its mass but not its volume. The relationship holds only up to about 30% fiber saturation. The apparent density does not change as the moisture increases beyond this point, and the volume remains the same. Like other species, the *Pinus sylvestris* wood density varies considerably between individual trees. Furthermore, wood with a moisture content between 0 and 30% can exchange water vapor with its environment, leading to changes in volume through swelling or shrinking [17].

### 2.3. Wood Modification by the HWE Method

**Figure 1:**
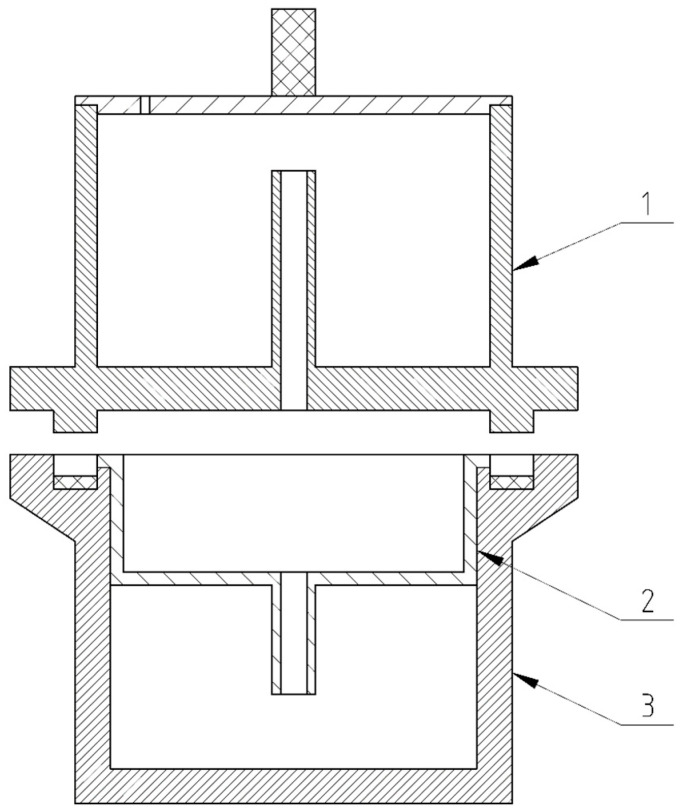
HWE set: (1) container for extraction solution; reactor lid; (2) container for chips; (3) reactor body.

The method used in this study is a novel HWE. There are no established methodological standards or commercial equipment for this specific application, making HWE unique. The HWE method [8] wood modification process used two wood fractions, *f1* (0 ÷ 1 mm) and *f2* (1 ÷ 4 mm). Wood fractions of 10 g were placed in separate containers with distilled water (400 g), and HWE was performed at 120 °C and 2 MPa for 30 min. The process was repeated six times for each fraction, with the extracted solution collected and the wood dried in between. The wet fraction was collected and analyzed, as well as the dry fraction, which consisted of suspended solids in water. The HWE set is presented in Figure 1.

The HWE 20 was collected in container No. 1. The material was placed in container No. 2, and a single HWE process was started after the reactor (element No. 1) was closed tightly. The new wood material and distilled water were replaced after HWE had been completed, and the process was repeated twice. The material and distilled water were again replaced after two repetitions, followed by three further extractions. The fractions were extracted six times. A compaction process was performed after the HWE process of the modified *Pinus sylvestris* material had been completed.

The acquisition and detailed analysis of the wet fraction of the material also played a significant role in the research, in addition to the modification of the material by the HWE method itself. The fraction in question, which consists of solid particles suspended in water, was obtained immediately after the HWE process. An evaporator separated the wet fraction from the solution to extract the valuable precipitate. By extracting precipitates and analyzing their properties, we aimed to obtain a deeper understanding of the HWE process by carefully examining the mass and properties. The technique allowed for the determination of the amount and type of substances leached from the wood and their potential for application in terms of antioxidants and biocide. Combined with the results from the fractions subjected to further treatment, the information obtained this way can be a valuable source of information for assessing the effects of HWE on the modified wood properties, chemical composition, and microstructure, as well as optimizing the HWE process parameters to suit the desired application.

### 2.4. Process of Material Compaction in the Prototype Compaction Chamber

Instron’s universal testing machine, model 3382, investigated the biocomposite mix’s mechanical properties and compaction. Compressive strength testing was performed according to the ASTM C365 standard [18]. The prepared samples were placed directly between the testing machine pressure plates and subjected to uniaxial compression. The machine precisely recorded the force and head displacement, enabling the determination of the material strength characteristics. Compaction studies of the wood biocomposite, in turn, were conducted on a custom-designed test stand, for which the testing machine was also a key component.

**Figure 2:**
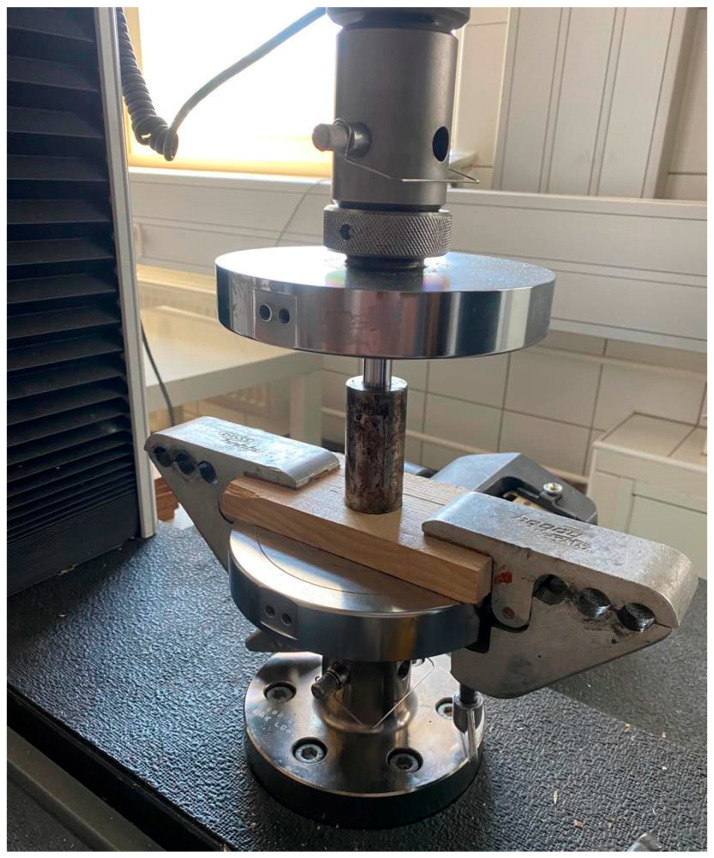
The compaction test set used to determine the level of compaction.

The compaction stand consisted of a compaction head, capable of exerting forces up to 100 kN via the Instron machine, and a heating module with integrated temperature control [19,20]. A dedicated test stand was necessary to research the compaction of the wood biocomposite. The stand had a testing machine, a unique compaction head, and auxiliary equipment. The comprehensive preparation of the stand enabled the tests to take place as planned. The compaction head and Instron testing machine collaborated during the experiments, producing a force of 100 kN. Compaction head pistons and chambers were chosen depending on the unit pressure on each piston, which was 3.5 MPa. The results suggest that the force applied was sufficient to compact the shredded material effectively. The compaction test set used to determine the level of compaction is presented in Figure 2.

Lignocellulosic materials require careful temperature control during compaction to achieve better binding and plasticization. The densification process was conducted in several stages. The compaction process was repeated thrice for each material to ensure repeatability. The samples were loaded in batches, one being loaded at a time. In the first step, shredded ABS material and a certain fraction of shredded material were added to the sleeve. The head was then heated in a dryer to a preset temperature along with the material. Ahead of this step, the plunger on the head of the testing machine was inserted into the machine, and the raw material was gradually compacted while the process parameters were controlled. Following completion, the plunger was moved back to its starting position, and a piston or additional tool was used to remove the product from the sleeve. The measurement and control of key parameters, such as the moisture content, granulometric composition of the material, and temperature, were made possible by the test stand. The resulting product was circular and cylindrical with a diameter of about 14 mm.

### 2.5. Ash Content

The ash content of *Pinus sylvestris* samples was determined through incineration in a laboratory muffle furnace (SNOL, Poland). During this process, the samples were heated at specific temperatures and for defined durations, ensuring complete combustion and leaving only the inorganic ash residue behind. The methodology for determining the ash content consisted of sequential steps: sample preparation, incineration, the weighing of the ash, and the calibration of the measuring equipment [21]. Before incineration, the samples were carefully dried to eliminate moisture, a critical step to ensure accurate results. Approximately two grams of pre-weighed *Pinus sylvestris* wood samples were placed into crucibles for each experimental cycle. The incineration process was conducted at a controlled temperature of 805 °C in an SNOL muffle furnace under high-vacuum conditions to ensure the precision of combustion. After approximately two hours, the burned samples were transferred to a desiccator to cool and prevent exposure to moisture. The weight of the ash in the crucible was then measured and used to calculate the ash content of the *Pinus sylvestris* wood samples [22,23]. This process provided valuable insights into the mineral composition of *Pinus sylvestris*, which is significant for understanding its applications in various industries.

The ash content of biomass is crucial because it can be used to determine whether the composition is inorganic, thereby influencing the properties of the final composite and its mechanical strength, flammability, and biodegradability. The study methodology prioritized precision, accuracy, and repeatability at every stage. The incineration conditions, such as the temperature and duration, were meticulously monitored to ensure consistency across all tests. The equipment, including the combustion furnace and analytical balance, was calibrated regularly to maintain the reliability of measurements. The ash content was expressed as a percentage of the initial sample weight or in grams per unit, enabling a detailed analysis of the *Pinus sylvestris* inorganic composition. These results have critical implications, particularly for bioenergy production, where mineral composition significantly determines the biomass efficiency and its suitability for renewable energy solutions. The *Pinus sylvestris* ash content data inform its potential applications in industrial processes that rely on specific mineral properties.

### 2.6. Statistical Analysis

Statistical analysis is vital to scientific research, determining significant differences between experimental groups and identifying the key factors influencing the observed results. Statistical analysis is based on variance, describing how data points are spread around the mean. Researchers can use it to track data distributions and variability so they can understand their findings’ consistency and reliability. Analyzing variance (ANOVA) is a statistical method widely used for studies involving more than two groups [24,25]. This method can compare multiple groups, considering both within-group and between-group variability. To assess the significance of differences between groups, variances among groups are compared with variances within groups. The variance between groups tends to be higher than within groups, indicating that the differences observed are likely caused by the experimental treatment or factor being investigated rather than chance.

The analysis of variance (ANOVA) is a statistical method for comparing the means of two or more groups. This method is based on the assumption that the data are typically distributed. Analyzing data with ANOVA can be powerful, but it does have limitations. For example, it is sensitive to outliers. Statistical outliers differ significantly from the other data points in the sample. There can be problems with the results if there are outliers in the data. The assumption of normal distribution is another limitation of ANOVA. The results of the ANOVA may be inaccurate if the data are not normally distributed. The compaction test data were analyzed using ANOVA. ANOVA was performed after checking the normality and variance of the data. The compaction work required for the different types of wood was significantly different according to the results of the ANOVA.

## 3. Results

### 3.1. Material Physical Properties

The research process required determining the appropriate fractions of *Pinus sylvestris* material to determine their durability and machinability. The wood fraction tested in the laboratory was within the *f1* (0 ÷ 1) range, while another fraction included shredded *Pinus sylvestris* with a size *f2* (1 ÷ 4). The moisture content of *Pinus sylvestris* material impacts its strength and physical properties, so controlling this parameter was essential during the tests. The *Pinus sylvestris* material used in this study had a moisture content of 12%, according to the research criteria. Two different mixtures were prepared according to the planned tests. The first mixture contained 50% ABS and 50% *Pinus sylvestris*, while the second included 50% ABS and 50% *Pinus sylvestris*. A compaction process was performed on each of these mixtures. To determine how particle size affects the process and mechanical properties of materials, *Pinus sylvestris f1* and *f2* fractions were compacted separately and then compared. The absolute moisture content of the different *Pinus sylvestris* fractions is presented in Table 1.

**Figure 3:**
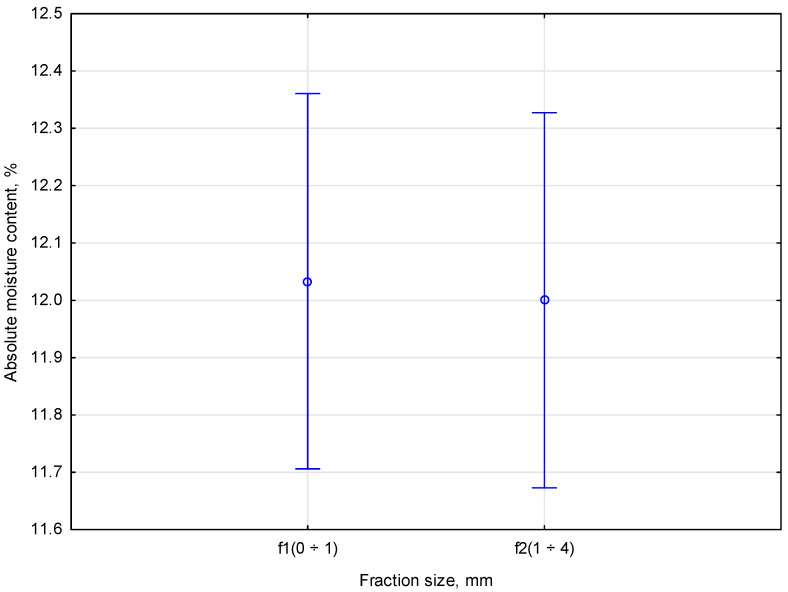
The average impact of the absolute moisture of the individual *Pinus sylvestris* fractions.

The measurement results for three replicates of the *f1* (0 ÷ 1) *Pinus sylvestris* fraction were 12.1%, 12.1%, and 11.9%, respectively. On the other hand, for the wood fraction *f2* (1 ÷ 4), the measurement results for three replicates were 12.2%, 11.7%, and 12.1%. The results indicated consistency in the *f1* (0 ÷ 1) and *f2* (1 ÷ 4) wood fractions. Approximately 12% of the samples from fractions *f1* (0 ÷ 1) and f2 (1 ÷ 4) had an absolute moisture content. The results of the tests were statistically analyzed by univariate ANOVA using the methodology. The average impact of the total moisture content on individual *Pinus sylvestris* fractions is presented in Figure 3.

Statistical analysis was performed on fractions *f1* and *f2* of the average absolute moisture content sample. The measured parameters showed no correlation in the statistical analysis. The statistics showed no correlation between the studied indicators, with *p* = 0.851 indicating statistical insignificance. *F*(1,4) = 0.04000 is the empirical value in this case. According to Duncan’s test, the groups had no significant differences.

### 3.2. Sludge After HWE

The HWE method was applied to the two fractions of shredded *Pinus sylvestris* wood. In HWE, compounds are extracted using water as a solvent, which penetrates the wood structure and, at elevated temperatures, facilitates the extraction of water-soluble compounds. This process partially degrades hemicelluloses, resulting in the formation of precipitates. The further degradation of these precipitates yields low-molecular-weight hemicelluloses and lignin. Approximately 10 g of wood chips was treated with HWE. Different stages of HWE can affect the mass and composition of the material. After treating the wood chips with 400 mL of distilled water in a specialized reactor, the solid residue mass obtained after evaporating the extract was determined. This residue, produced by evaporating the water, is presented in Table 2.

**Figure 4:**
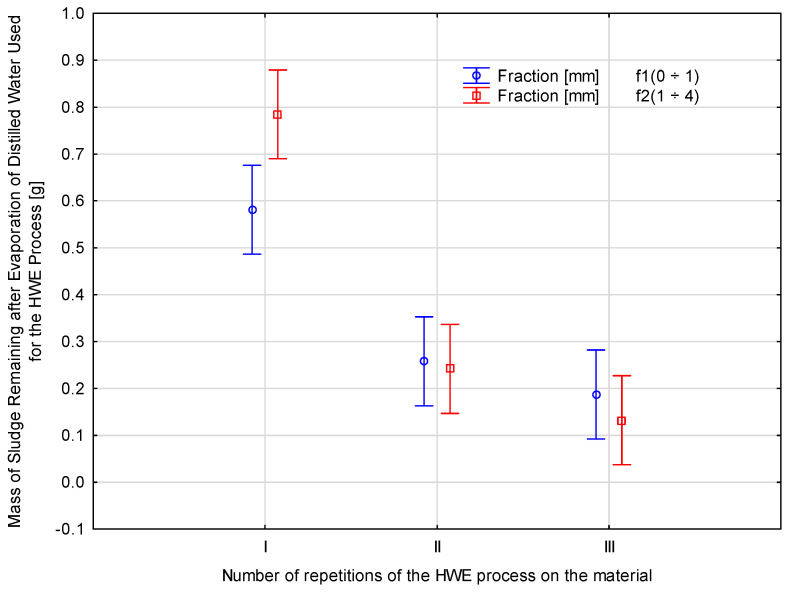
Marginal mean relationships between the values of the mass of the sludge remaining after the evaporation of the distilled water used in the HWE process depending on the fraction size and the number of repetitions.

The study focused on the remaining sludge after evaporating distilled water during water extraction (HWE). Fractions were created and subjected to three HWE processes, each consisting of three repetitions, and values were calculated for each fraction. After the third extraction cycle, *f2*(1 ÷ 4) showed the least sludge. As a result, a large amount of sludge developed in the same fraction *f2*(1 ÷ 4). The reduction in the amount of sediment in each fraction during the HWE cycles was estimated; this indicated that sediment levels limit *Pinus sylvestris* extraction. The graphic analysis of the statistical analysis by ANOVA of the main effects, including the mass of residue left after the evaporation of distilled water in HWE, depending on the size and number of repetitions, is presented in Figure 4.

The fraction size was compared with the number of repetitions of the HWE process to determine the statistical relationship between the fraction size and number of extractions. The study results were analyzed using statistical analysis, comparing the effects of different parameters and determining their correlation. Three homogeneous groups were selected when no statistically significant difference existed between the measured parameters. An analysis of *p*-values indicated that the measurement was significant at *p* = 0.02462. The *p*-values for all the ANOVA tests were below 0.05, showing that wood types require different compaction levels. According to the ANOVA tests, there were 2 and 12 degrees of freedom, respectively. Based on the ANOVA test, we obtained a F-statistic of 5.229. Three homogeneous groups were identified based on Duncan’s test. In the first group, the *f1* fraction was included. In the second, the *f2* fraction and ABS were included in the third.

### 3.3. Compression Process with Energy Characteristics

#### 3.3.1. Analysis of Energy Consumption in the Compaction Process

**Figure 5:**
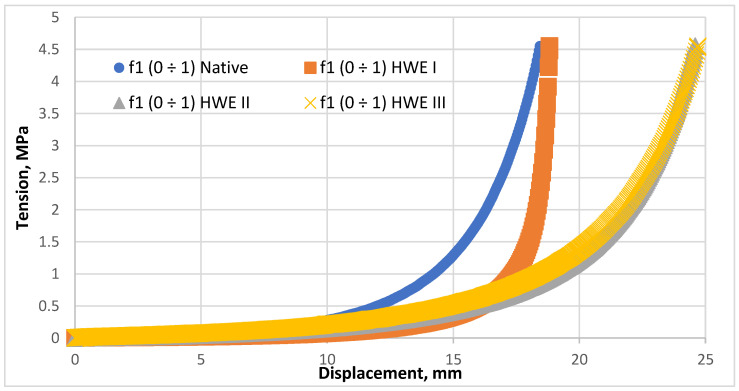
Compaction characteristic of the concentration process for fraction *f1*(0 ÷ 1).

The compaction of the wood biocomposite was performed on a dedicated test stand using an Instron tester and a unique compaction head. According to the methodology, the force applied was enough to compact the material adequately. The compaction process involved the introduction of material into the compaction sleeve and gradual compaction under pressure. The compaction process was repeated three times for each material, and the average of these replicates is presented. The compaction of two *Pinus sylvestris* fractions, *f1*(0 ÷1) and *f2*(1 ÷ 4), was performed without additional material. The HWE process was applied before the compaction of these fractions. A study showed that the attempt to compact *Pinus sylvestris* wood fractions *f1*(0 ÷ 1) and *f2*(1 ÷ 4) after HWE at 22 °C did not yield the expected results since it failed to produce a uniform and consistent product. Several pieces of material were scattered after the compressed material left the chamber. The characteristics of the compaction process for fraction *f1*(0 ÷ 1) with different modifications are presented in Figure 5.

The laboratory tests revealed that, for each raw material, the initial displacement increased slowly with pressure. After removing air during the kneading process, the material was mechanically kneaded further to eliminate any remaining air pockets between the particles. This stage required minimal force, which was below the detection limit of the testing equipment. For the *f1*(0 ÷ 1) HWE I material, the displacement stabilized at 18 mm after an initial increase in pressure. At 4.5 MPa, the *f1*(0 ÷ 1) HWE I material tended to compact more similarly to the *f1*(0 ÷ 1) negative material. The compaction results for HWE II and III of *f1*(0 ÷ 1) were similar. The displacement reached 24.6 mm at 4.5 MPa, but it is possible that further pressure increases could result in more significant displacement.

For a deeper understanding of fraction effects, analyses were performed using the energy value of the compaction process after the HWE treatment and the native form. An analysis of the total work value of the compaction process can provide the specific parameters used to determine the total work value. To describe the compaction of a material, a third-degree polynomial is fitted to an arithmetic function (y = ax3 + bx2 + cx + d). The Gauss–Newton least squares method was used to develop an estimated model. A mathematical model with an R2 coefficient of determination greater than 0.95 must fit the analysis well. With the specified parameters, the integral equation, coefficients, total compaction value, and how the coefficients were evaluated can be found in Table 3.

**Figure 6:**
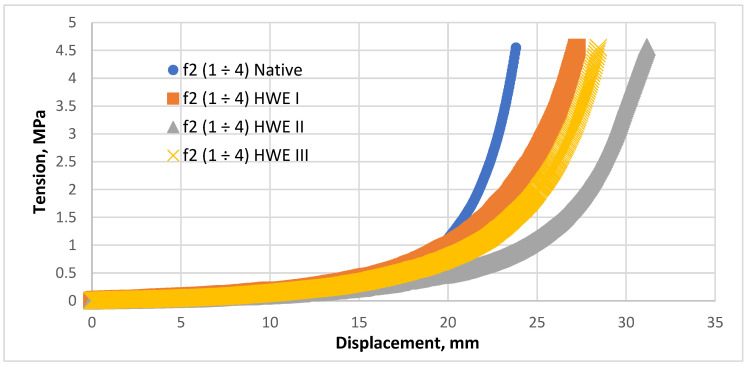
Compaction characteristic of the concentration process for fraction *f2*(1 ÷ 4).

A further investigation of the thickening process focused on the *f2* (1 ÷ 4) fraction. Similar to the previous results, this *Pinus sylvestris* fraction laboratory testing showed a slow pressure increase with increasing displacement. The analysis suggests that for HWE I, HWE II, and HWE III materials, displacement could continue to grow beyond the 4.5 MPa pressure limit of the test. The *f2* (1 ÷ 4) HWE II material exhibited the highest level of compaction. Comparing the compaction behavior of the hot-water-extracted material with the untreated material, it can be concluded that the HWE process increased the material compressibility. The compaction characteristic of the concentration process for fraction *f2* (1 ÷ 4) is presented in Figure 6.

**Figure 7:**
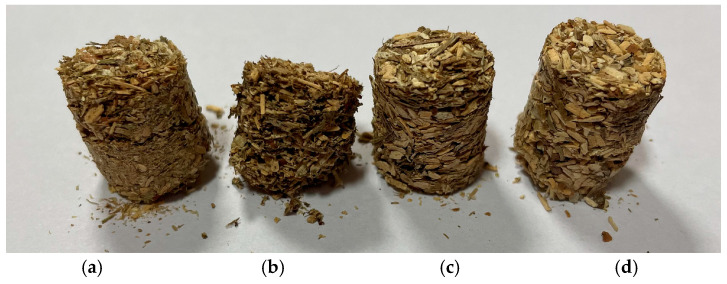
Material: (**a**) *Pinus sylvestris* fraction *f1*(0 ÷ 1)—Native—after compaction without HWE; (**b**) *Pinus sylvestris* fraction *f1*(0 ÷ 1) after HWE I; (**c**) *Pinus sylvestris* fraction *f1*(0 ÷ 1) after HWE II; (**d**) *Pinus sylvestris* fraction f1(0 ÷ 1) after HWE III.

The results indicate that the HWE process significantly influences the final compaction of the material. By removing organic substances such as resins and volatiles, HWE increases the number of available bonding sites during compaction. Additionally, HWE modifies the structure of wood fibers, altering their physical and chemical properties and thus enhancing their compressibility. While HWE facilitates densification, it can also affect the final properties of the material, such as its strength and resistance to environmental conditions. Further research into the mechanisms of HWE and its effects on the structure and properties of the material is needed to gain a deeper understanding of its benefits for composite manufacturing. Figure 7 shows samples of the *f1* (0 ÷ 1) *Pinus sylvestris* fraction after compaction, both with and without (Native). The HWE process is presented in Figure 7.

The relationship between the calculated energy value of the compaction process and the *f2*(1 ÷ 4) fraction was further investigated. The total work can be determined using specific parameters in a compaction study. A third-degree polynomial is commonly used to describe the relationship between pressure and displacement during material compaction. A least-squares model was employed to calculate the trendline. The model coefficient of determination (*R²*) did not exceed 0.95. The integral equation, the coefficients of the polynomial, and the total compaction work for each parameter are presented in Table 4.

The use of a third-degree polynomial to describe the forces occurring during the compaction process was indicated by statistical analysis. A second and seventh-degree polynomial was necessary in two cases to match functions that followed linear paths. The coefficient of determination R2 for this example was estimated at 0.95. To calculate the total work for the material compaction process, we determined the change in the compaction force over time for native and HWE materials. Determining how much material was compressed is possible by calculating the packing factor with distilled water and the material density. The values of the total compaction work for fractions *f1* and *f2* are presented in Table 5.

The study investigating the density and compaction behavior of two *Pinus sylvestris* fractions (*f1* and *f2*) after different hydrothermal water extraction (HWE) treatments (HWE I, HWE II, HWE III) and in their native state revealed that HWE I produced the highest volumetric and specific density values. For the *f1* fraction, the density reached 181.08 kg/m³, while the *f2* fraction reached 250.69 kg/m³ after HWE I. Both fractions exhibited the lowest densities in their native state: 147.19 kg/m³ for *f1* and 203.77 kg/m³ for *f2*. Interestingly, the highest density achieved through compaction was observed in the native state for *f1* (241.79 kg/m³), while for *f2*, the highest density was achieved after the HWE I treatment (217.04 kg/m³). The lowest compaction work was required for both fractions in their native state: 1.40 × 10⁻⁵ J for *f1* and 2.13 × 10⁻⁵ J for *f2*. The highest compaction work was required for *f1* after HWE I (2.07 × 10⁻⁵ J) and *f2* after HWE II (2.71 × 10⁻⁵ J). The HWE method increases both fractions’ density relative to their natural states but requires more compaction. Energy consumption during processing needs to be minimized while minimizing this trade-off.

#### 3.3.2. Energy Expenditure in the Biocomposites Compaction Process

This study aimed to gather data to calculate the energy dynamics of the compaction process. The analysis focused on the work required to compact different wood/ABS mixtures. It also explores the interplay between these factors and their combined influence on energy requirements. This analysis aims to identify strategies for optimizing energy efficiency in biocomposite production by examining these variables, contributing to the development of more renewable manufacturing practices. The total compaction work for mixtures of selected *Pinus sylvestris* fractions with 50% ABS docking is shown in Table 6

**Figure 8:**
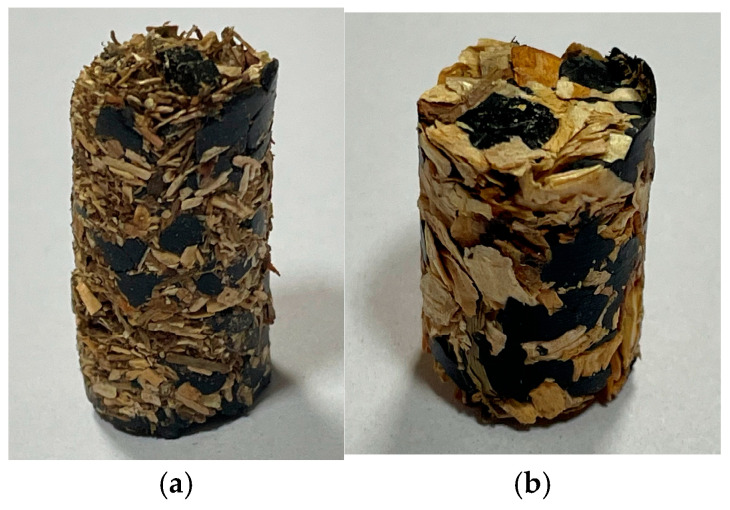
Prepared biocomposites: (**a**) *f1*, ABS 50%, (**b**) *f2*, ABS 50%.

An analysis of the total compaction work for *Pinus sylvestris* mixtures with 50% ABS plastic (fractions *f1* and *f2*) reveals a significant difference depending on the *Pinus sylvestris* wood fraction used. Despite the similar specific density of the ABS plastic in both mixtures (826.66 kg·m^−3^), the total compaction work for the mixture containing the *f1* fraction and ABS (1.95 × 10⁻⁵ J) is substantially higher than that for the mixture containing the *f2* fraction and ABS (4.20 × 10⁻⁷ J). This difference may be attributed to the distinct properties of the *f1* and *f2 Pinus sylvestris* fractions, suggesting that the *f1* fraction requires more energy to compact. Using the f2 fraction in ABS blends could lead to energy savings in the production of biocomposite materials. The prepared biocomposites, *f1* and *f2*, with 50% ABS are presented in Figure 8.

The size of the wood particles used in the two biocomposites is the most noticeable difference. The first biocomposites incorporate a finer fraction of 0–1 mm particle sizes. The second includes a coarser fraction of wood, with particle sizes between 1 and 4 mm. Particle size differences impact material homogeneity. Due to the coarse wood fraction, a nonhomogeneous appearance is created by the ABS polymer matrix; a more homogeneous material results from the smaller particle size in the second biocomposite. In addition to their density and compaction work, these two biocomposites differ. The density of the biocomposite with a coarser wood fraction is 214.37 kg/m^3^, requiring more compaction work (2.71 × 10^−5^ J). The biocomposite with a finer wood fraction has a 198.12 kg/m^3^ density and requires compression work of 1.92 × 10^−5^ J. As a result of these differences, the particle size impacts the final properties and processing requirements of biocomposites.

### 3.4. Ash Content Analysis

**Figure 9:**
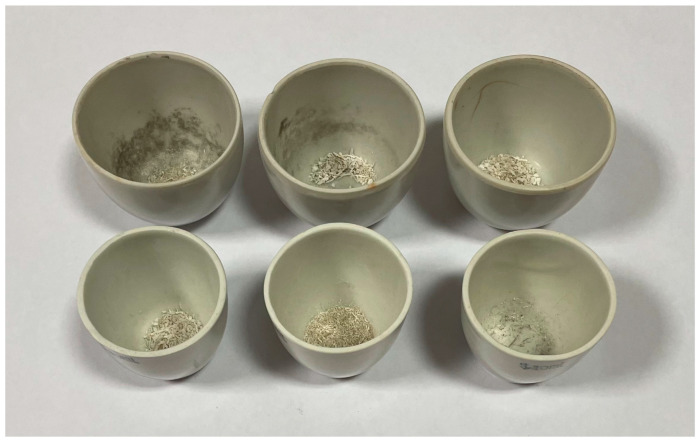
Examples of ash samples used for examination.

To determine the ash content of the test material, the sample had to be ash at high temperatures. This was followed by the accurate weighing of the residue. The ash content may reflect the densification process (brittle material), as stated in the literature [20]. The samples were burned at 800 °C in a SNOL muffle furnace. The residual ash was then weighed using an analytical balance to determine the ash content. Examples of the ash samples used for examination are presented in Figure 9.

This standard method, commonly used in chemical and physical analyses, ensured the accuracy of the results. Nine samples were prepared for analysis: three ABS samples, three samples of *Pinus sylvestris* fraction *f1*(0 ÷ 1), and three samples of *Pinus sylvestris* fraction *f2*(1 × 4). After burning, the samples were cooled in a desiccator and weighed. This process, which determines the ash content of a material, is crucial for analyzing its chemical composition. It is essential to control process parameters such as the temperature and duration to ensure uniform burning conditions. Variations in the consistency or density of different material fractions can influence the burn process and the final ash composition despite similarities in their chemical makeup. The results of the ash content analysis are presented in Table 7.

**Figure 10:**
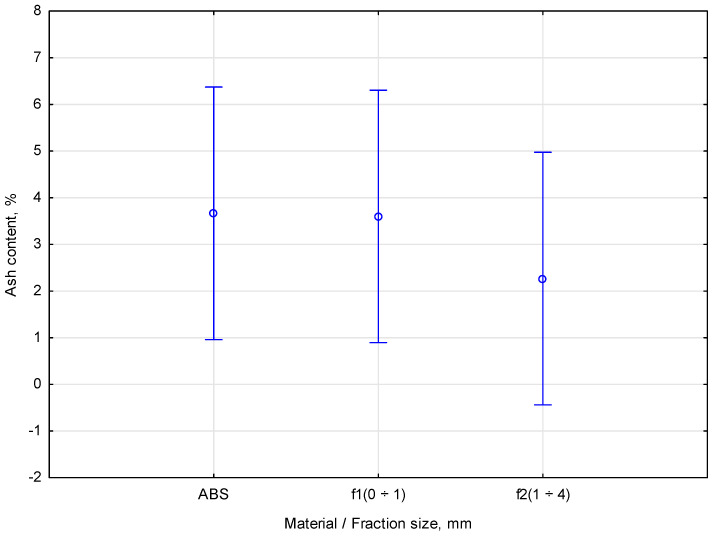
Statistical relationship between material and ash content.

An analysis of the ash masses resulting from the combustion of three different materials was conducted. The materials included ABS *f1* (0 ÷ 4), wood fraction f1 (0 ÷ 1), and wood fraction *f2* (1 ÷ 4). The ABS sample produced ash masses of 0.004 g, 0.045 g, and 0.061 g, with an average of 0.037 g. The *Pinus sylvestris* wood fractions *f1*(0 ÷ 1) yielded ash masses of 0.333 g, 0.037 g, and 0.038 g, averaging 0.136 g. The wood *f2*(1 ÷ 4) sample resulted in ash masses of 0.012 g, 0.016 g, and 0.040 g, with an average of 0.023 g. The analysis showed that *Pinus sylvestris* wood fraction *f1*(0 ÷ 1) produced the most ash (0.136 g), followed by ABS (0.037 g) and then wood fraction *f2*(1 ÷ 4) (0.023 g). This suggests that larger wood pieces (*f2*) burn with less ash than smaller ones (*f1*). The most considerable ash amount was generated from wood fraction *f1*(0 ÷ 1). A graphical representation of the univariate ANOVA analysis of the ash content for the different materials, illustrating the relationship between the material type and the resulting ash content, is presented in Figure 10.

A further statistical analysis of *Pinus sylvestris* wood fractions f1 and f2 and the ABS thermoplastic showed no correlation between the examined parameters. Duncan’s test evaluated differences in the ash content (%) between the materials, considering the material fractions. The test revealed no statistically significant differences. The significance level was *p* = 0.62437, and the intergroup mean squared error was estimated at 3.6689 with 6 degrees of freedom. The test identified three statistically homogeneous groups. The material of fraction f2 had an average ash content of 2.26%. The f1 fraction had an average ash content of 3.6%. The ABS material had an average ash content of 3.67%. Each material was assigned to the same group, suggesting their statistical similarity.

## 4. Discussion

The HWE method was used to investigate the compaction behavior of two *Pinus sylvestris* wood fractions, *f1*(0 ÷ 1) and *f2* (1 ÷ 4), for use in biocomposite production with ABS plastic. Initial compaction tests at 22 °C produced unsatisfactory results since the material failed to form a cohesive structure. Based on the existing literature on biomass compaction, it is evident that temperature plays a crucial role in achieving desirable material properties. Compared with the compacted rapeseed straw, Pietrzak and Górski [26] observed that higher temperatures improved consistency and uniformity, while Lisowski [27] observed an increased density and strength. According to our findings, insufficient thermal energy could result in suboptimal compaction at 22 °C due to ineffective bonding and densification.

The compaction of *Pinus sylvestris* fractions at elevated temperatures should be explored to determine the optimal processing conditions. Previous studies have shown that temperature significantly affects the compaction behavior of *Pinus sylvestris* wood. Similar results were found in a study on the compaction of beech wood [28]. Research suggests that temperature significantly influences the mechanical properties of materials, including their flexural and compressive strength. This may explain the inability to compact *Pinus sylvestris* at 22 °C [29]. The study showed that *f1*(0 ÷ 1) fractions required less energy when compacted at 22 °C than *f2*(1 ÷ 4). The particle size may affect the energy efficiency of the compaction process. Energy consumption may also differ depending on the moisture content [30] or the type of HWE process [31]. Therefore, it is necessary to conduct further research to understand the energetics of compaction under various particle sizes fully.

Besides temperature, moisture content plays a vital role in wood compaction. The moisture content can affect a material’s plasticity and susceptibility to deformation, affecting the compaction process energy efficiency. According to a study, *Pinus sylvestris* wood with a high moisture content is more susceptible to compaction [32]. A survey of beech wood compaction [33] reached similar conclusions, finding that a decrease in the moisture content decreased the force required to compact the material. In the present study, the moisture content was not directly examined regarding compaction, but further studies should consider this factor.

The ash content provides valuable insights into the chemical composition of biocomposite materials and its potential effects on the properties of the final product, such as its mechanical strength, flammability, and biodegradability [23,34]. Using the methodology described in [23], we demonstrated that it is possible to determine the ash content of *Pinus sylvestris* wood by incineration at about 800 °C. To assess the impact of the HWE process on ash content, the tested *Pinus sylvestris* fractions were compared to those in the existing literature. The amount of ash in wood differs significantly depending on factors such as the genotype, growth conditions, tree age, and anatomical part [11]. The ash content of *Pinus sylvestris* wood was reported in the literature [35], revealing that the ash content differed between different tree species, including black *Pinus nigra* and Calabrian *Pinus brutia*, showing that at a species level and tree section level, the ash content differed significantly. According to the latter study [22], the wood ash content was determined using modern analytical techniques.

The chemical composition and possible effects of HWE on the properties of the biocomposite can be determined from studies on the impact of HWE on the ash content of lignocellulosic material [2]. The present study found that *Pinus sylvestris* samples treated with HWE III contained less ash than their non-extracted (Native) counterparts. It appears that HWE can be helpful in modifying lignocellulosic fibers, improving performance, and expanding their industrial applications. The ash content can be affected by HWE depending on the plant species and extraction process parameters. During the extraction of *Pinus sylvestris* wood, the ash content was reduced, which the authors attribute to mineral substances leaching from the material. A study on *Miscantus Gigantus* [36] also concluded that HWE removes certain minerals, including potassium and silicon. To achieve this, additional studies must be conducted to fully understand the effect of HWE on the ash content of different plant species and to optimize the extraction process parameters.

This study has several limitations in terms of its statistical analysis. In the first place, the sample size was relatively small. As a result, the statistical tests may not have had the power to detect significantly different results between the groups. There was also a certain amount of variation in the data. The results of the statistical analysis may also have been affected by this. The findings of this study should be confirmed through further research with a larger sample size and less variable data.

## 5. Conclusions

The main focus of this study was to investigate how HWE impacts the energy dynamics of biocomposite production using wood and ABS. HWE treatment was found to alter the physical and chemical properties of *Pinus sylvestris*, influencing its suitability for biocomposite production. The HWE method increased the material density by removing extractives and modifying the wood structure. This increase in density translates to a higher density in the final biocomposites, which is advantageous due to its improved mechanical properties. The effect of HWE on energy consumption during compaction varied. While specific HWE treatments reduced the energy required, others resulted in a slight increase or no significant change.

This research aimed to investigate how HWE affects the energy efficiency of biocomposite production using wood and acrylonitrile butadiene styrene (ABS) plastic. It aimed to determine whether HWE treatment could improve the energy efficiency of biocomposite production and to identify optimal HWE treatment parameters for achieving this target. Based on the results of this study, HWE treatment can indeed improve the energy efficiency of composite production, and the optimal HWE treatment parameters depend on the wood and ABS plastic used.

Optimizing energy efficiency necessitates the careful consideration of the particle size, moisture content, and specific HWE treatment parameters. Previous research indicated that the f2 fraction (larger particle size) requires more energy for compression than the f1 fraction, suggesting that particle size plays a role in the energy dynamics of biocomposite production. Further studies are warranted to explore the complex interactions between the factors influencing energy consumption during compaction and to assess the long-term properties of biocomposites made with HWE-treated *Pinus sylvestris*. These findings suggest that HWE can improve the efficiency of ABS and *Pinus sylvestris* composites, but it is crucial to acknowledge and address the environmental impact associated with their use.

## Figures and Tables

**Table 1:** The absolute moisture content of individual *Pinus sylvestris* fractions.

Material	Mass of Vessel, g	Mass of Vessel with the Material, Before Drying, g	Mass of Vessel with the Sample After Drying It to a Constant Weight, g	Sample Absolute Moisture, %	Average of Absolute Moisture, %
*Pinus sylvestris* fraction *f_1_* (0 ÷ 1)	51.512 51.509 51.503	61.515	52.278	12.1	12 ^a^
		61.530	52.273	12.1	
		61.514	52.282	11.9	
*Pinus sylvestris* fraction *f_2_* (1 ÷ 4)	79.012 79.009 79.013	89.044	79.771	12.2	12 ^a^
		89.020	79.798	11.7	
		89.094	79.782	12.1	

^a^—homogeneous group.

**Table 2:** Mass of sludge in HWE process after evaporation of distilled water.

Fraction, mm	Number of Repetitions of the HWE Process	Mass of Sludge Remaining After Evaporation of Distilled Water Used in the HWE Process, g	Average Mass of Sludge Remaining After Evaporation of Distilled Water Used in the HWE Process, g
*f_1_* (0 ÷ 1)	I	0.511	0.581 ^a^
		0.661	
		0.572	
	II	0.303	0.258 ^b^
		0.356	
		0.115	
	III	0.211	0.187 ^b^
		0.192	
		0.159	
*f_2_* (1 ÷ 4)	I	0.701	0.785 ^c^
		0.891	
		0.762	
	II	0.221	0.242 ^b^
		0.213	
		0.292	
	III	0.114	0.132 ^b^
		0.132	
		0.151	

^a,b,c^—homogeneous groups.

**Table 3:** Calculation of total compaction work using integral equation.

Total Work Done Under Specified Conditions W_(τ, φ)_	Coefficient of Determination R^2^	Displacement l, mm	Total Compaction Operation, J
$W_{\left(f_{1},HWE\;I\right)}=\int_{0}^{0.001\cdot l}\left(5\times 10^{-6}x^{6}-3\times 10^{-4}x^{5}+4.4\times 10^{-3}x^{4}-0.341x^{3}+0.113x^{2}-0.119x\right)dx$	0.960	18.81	2.07 × 10^−5^
$W_{\left(f_{1},HWE\;II\right)}=\int_{0}^{0.001\cdot l}\left(0.0007x^{3}-0.0128x^{2}+0.0637x\right)dx$	0.975	24.59	1.92 × 10^−5^
$W_{\left(f_{1},HWE\;III\right)}=\int_{0}^{0.001\cdot l}\left(0.0006x^{3}-0.012x^{2}+0.0659x\right)dx$	0.983	24.70	2.00 × 10^−5^
$W_{\left(f_{1},\;Native\right)}=\int_{0}^{0.001\cdot l}\left(0.0016x^{3}-0.0232x^{2}+0.0829x\right)dx$	0.981	18.44	1.40 × 10^−5^

**Table 4:** Calculate total compaction work using the integral equation.

Total Work Performed Under Specified Conditions *W*_(*τ*, *φ*)_	Coefficient of Determination *R*^2^	Displacements l, mm	Total Compaction Operation, J
$W_{\left(f_{2},HWE\;I\right)}=\int_{0}^{0.001\cdot l}\left(0.0005x^{3}-0.01x^{2}+0.0614x\right)dx$	0.988	27.23	2.27 × 10^−5^
$W_{\left(f_{2},HWE\;II\right)}=\int_{0}^{0.001\cdot l}\left(0.0003x^{3}-0.0088x^{2}+0.056x\right)dx$	0.970	31.17	2.71 × 10^−5^
$W_{\left(f_{2},HWE\;III\right)}=\int_{0}^{0.001\cdot l}\left(0.0004x^{3}-0.009x^{2}+0.0542x\right)dx$	0.980	28.44	2.19 × 10^−5^
$W_{\left(f_{2},Native\right)}=\int_{0}^{0.001\cdot l}\left(0.0008x^{3}-0.0164x^{2}+0.0755x\right)dx$	0.954	23.82	2.13 × 10^−5^

**Table 5:** Total values of the compaction work for fraction *f1* and fraction *f2*.

Fraction	Average Volume Density (SD) ^1^, kg·m^−3^	Average Density Calculated as Specific Density (Inner and Outer Pores), kg·m^−3^	Average Density After Compaction (SD) ^1^, kg·m^−3^	Total Compaction Operation, J
*f_1_*, *HWE I*	181.08 (1.23)	250.69 (1.70)	222.08 (4.35)	2.07 × 10^−5^
*f_1_*, *HWE II*	149.50 (1.96)	206.97 (2.71)	198.12 (4.49)	1.92 × 10^−5^
*f_1_*, *HWE III*	164.24 (1.18)	227.37 (1.63)	215.33 (4.45)	2.00 × 10^−5^
*f_1_*, *Native*	147.19 (1.72)	203.77 (2.38)	241.79 (4.50)	1.40 × 10^−5^
*f_2_*, *HWE I*	155.81 (0.87)	215.70 (1.20)	217.04 (4.52)	2.27 × 10^−5^
*f_2_*, *HWE II*	145.29 (1.61)	201.14 (2.23)	214.37 (4.47)	2.71 × 10^−5^
*f_2_*, *HWE III*	147.39 (1.29)	204.05 (1.78)	205.46 (4.44)	2.19 × 10^−5^
*f_2_*, *Native*	126.45 (0.40)	175.06 (0.55)	208.28 (4.53)	2.13 × 10^−5^

^1^ SD—standard deviation.

**Table 6:** Total values of compaction work for mixtures of selected *Pinus sylvestris* fractions with 50% ABS added.

Mix	Average Density Calculated as Specific Density (Inner and Outer Pores), kg·m^−3^	ABS Density (SD) ^1^, kg·m^−3^	Specific Density ABS (SD) ^1^, kg·m^−3^	Total Compaction Operation, J
*f_1_*, *ABS 50%*	203.77 (2.38)	826.66 (1.82)	265.95 (3.77)	1.95 × 10^−5^
*f_2_*, *ABS 50%*	175.06 (0.55)	826.66 (1.82)	266.21 (6.55)	4.20 × 10^−7^

^1^ SD—standard deviation.

**Table 7:** Ash content.

Material	Weight of Empty Containers, g	Material Weight, g	Weight After Combustion (Container + Material), g	Ash Mass, g	Ash Content, %
ABS	55.934 58.308 31.750	2.011	55.938	0.004	0.4
		2.002	58.353	0.045	4.5
		2.011	31.811	0.061	6.1
*Pinus sylvestris* fraction *f1*(0 ÷ 1)	50.44 31.110 29.519	2.006	50.773	0.033	3.3
		2.007	31.147	0.037	3.7
		2.020	29.557	0.038	3.8
*Pinus sylvestris* fraction *f2*(1 ÷ 4)	33.143 59.575 57.719	1.755	33.155	0.012	1.2
		1.807	59.591	0.016	1.6
		2.005	57.759	0.040	4.0

## Data Availability

The original contributions presented in this study are included in the article. Further inquiries can be directed to the corresponding author.
